# Complete Genome Sequence of the Aerobic Facultative Methanotroph Methylocella tundrae Strain T4

**DOI:** 10.1128/MRA.00286-19

**Published:** 2019-05-16

**Authors:** Martine A. R. Kox, Muhammad Farhan Ul Haque, Theo A. van Alen, Andrew T. Crombie, Mike S. M. Jetten, Huub J. M. Op den Camp, Svetlana N. Dedysh, Maartje A. H. J. van Kessel, J. Colin Murrell

**Affiliations:** a Department of Microbiology, IWWR, Radboud University, Nijmegen, The Netherlands; b School of Environmental Sciences, University of East Anglia, Norwich, United Kingdom; c School of Biological Sciences, University of East Anglia, Norwich, United Kingdom; d Winogradsky Institute of Microbiology, Research Center of Biotechnology of the Russian Academy of Sciences, Moscow, Russia; University of Southern California

## Abstract

Methylocella tundrae T4^T^ is a facultative aerobic methanotroph which was isolated from an acidic tundra wetland and possesses only a soluble methane monooxygenase. The complete genome, which includes two megaplasmids, was sequenced using a combination of Illumina and Nanopore technologies. One of the megaplasmids carries a propane monooxygenase gene cluster.

## Abstract (precis)

Methylocella tundrae T4^T^ is a facultative aerobic methanotroph which was isolated from an acidic tundra wetland and possesses only a soluble methane monooxygenase. The complete genome, which includes two megaplasmids, was sequenced using a combination of Illumina and Nanopore technologies.

## ANNOUNCEMENT

Methane-oxidizing bacteria (MOB) play a major role in the global conversion of methane, since they utilize methane as a source of carbon and energy. MOB are widespread in nature, especially in methane-rich areas (1–3). Most MOB are aerobic Gram-negative bacteria belonging to the *Alphaproteobacteria*, *Gammaproteobacteria*, or *Verrucomicrobia*. MOB are mostly obligate one-carbon utilizers, except for *Methylocella* species, Methylocapsa aurea, and some *Methylocystis* species, which also utilize multicarbon compounds (4–8). Unlike most methanotrophs, *Methylocella* species rely entirely on soluble methane monooxygenase (sMMO) for methane oxidation and lack particulate methane monooxygenase (pMMO). The draft genome sequences of two *Methylocella* strains Methylocella silvestris BL2^T^ (9) and Methylocella silvestris TVC, have been published (10). We now report the complete genome sequence of Methylocella tundrae T4^T^, isolated from an acidic *Sphagnum* tundra peatland in northern Russia (11).

*M. tundrae* T4^T^ was cultivated on M2 agar medium (12) with methane (10% [vol/vol] in the headspace) as the sole carbon and energy source. Multiple colonies were harvested, from which genomic DNA was extracted using the ammonia acetate extraction method (13). Sequencing was performed using a dual sequencing strategy. First, the DNA was sequenced using the MinION access program (Oxford Nanopore Technologies, Oxford, UK). The library was prepared using kit number SQK-LSK108 with the fragmentation step using a g-TUBE (2 × 60 s at 5,000 rpm; Covaris, Inc., Woburn, MA, USA) and subsequently sequenced using a FLO-MIN106 R9.4.1 flow cell. Next, DNA was also sequenced using paired-end (2 × 150-bp) Illumina MiSeq sequencing to obtain high-quality sequences. The library was prepared using the Nextera XT kit (Illumina, San Diego, CA, USA) and sequenced using the MiSeq reagent kit v3 (Illumina).

A total of 6,386,172 raw Illumina paired-end reads (mean length, 149 bp) were obtained, subsequently trimmed using default settings, with a minimum read length of 100 bp and trimming of the first 15 bp, and merged in CLC Genomics Workbench v11 (Qiagen Aarhus A/S, Denmark). Nanopore sequencing yielded 49,146 raw reads (mean length, 6,466 bp; N_50_, 8,738 bp), which were base-called using Albacore v2.1.10, assembled using Canu v1.8 (14), and polished first with Racon v1.3.1 (15) and then with Illumina reads using Pilon v1.23 (16), all with default settings. This resulted in three circular scaffolds. Circularity was further investigated using Repseek v6.6 with default settings (17), which showed overlap (>97% identity for >8,800 bp) at the start and end of every scaffold. Additionally, it was confirmed that there was no overlap between the different scaffolds by calculating dot plots with Gepard v1.40 using default settings (18). Genome completeness was checked with CheckM v1.0.12 (completeness, 98.35%; contamination, 0.73%) (19). Finally, the genome was annotated via the MicroScope platform (20). The largest scaffold consisted of the chromosome (Table 1), and the smaller two scaffolds represented two megaplasmids (Table 1, megaplasmids A and B), each with their own alphaproteobacterial plasmid replication site (*repABC* operon) (21). This is the first time megaplasmids have been observed in *Methylocella* species.

**TABLE 1:** Genomic attributes of the 3 genetic elements that make up the genome of Methylocella tundrae T4^T^

	Data for:		
Attribute	Chromosome	Megaplasmid A	Megaplasmid B
Size (bp)	3,909,804	303,933	208,729
DNA G+C content (%)	61.80	61.84	61.46
No. of DNA scaffolds	1	1	1
Circular	Yes	Yes	Yes
Total no. of genes^*a*^	4,276	390	275
Protein coding density (%)	86.71	85.56	87.08
No. of RNA genes	59	0	0
No. of rRNA genes	2 (5S,16S, and 23S)	0	0
No. of tRNA genes	53	0	0
No. of pseudogenes	61	13	2
No. of genes with function predicted	616	53	15
No. of genes assigned to COGs^*b*^	2,957	252	96
Coverage (×)	102	85	114
GenBank accession no.	LR536450	LR536451	LR536452

a Without artifacts.

b COGs, Clusters of Orthologous Groups.

The presence of all genes required for the formation and functioning of sMMO (*mmoXYBZDCRG*) and the absence of pMMO genes was confirmed. Gene operons encoding calcium-dependent methanol dehydrogenase (*mxaFJGIRSACKLD*) and lanthanide-dependent methanol dehydrogenase (*xoxFJG*) are also present. Like Methylocella silvestris BL2^T^, all genes required for carrying out the complete oxidation and assimilation (via the serine cycle) of formaldehyde were identified. Genes related to nitrogen metabolism are located on the chromosome, including *nifHDK* (N_2_ fixation), *narGHJI* (membrane-bound respiratory nitrate reduction), and *nosRZDFY* (nitrous oxide reductase). Interestingly, the propane monooxygenase gene cluster (*prmABCD*) is located on megaplasmid A. Megaplasmid B contains mainly genes of unknown function. Further genome analyses and detailed comparative genomics studies of the growing number of *Methylocella* species are required to better understand the phylogeny and evolution of facultative methanotrophy in these unique bacteria.

### Data availability.

This whole-genome sequencing project has been deposited in the ENA within project number PRJEB31709. The raw paired-end Illumina reads were deposited with SRA accession number ERR3223707 and the raw MinION reads with SRA number ERR3224043. The assembled genome is available under GenBank accession numbers LR536450 to LR536452. The version described in this paper is the first version.
